# Comparison of similarity-based tests and pooling strategies for rare variants

**DOI:** 10.1186/1471-2164-14-50

**Published:** 2013-01-24

**Authors:** Sergii Zakharov, Agus Salim, Anbupalam Thalamuthu

**Affiliations:** 1 Human Genetics, Genome Institute of Singapore, 60 Biopolis Street, Singapore 138672, Singapore; 2 Saw Swee Hock School of Public Health, National University of Singapore, 16 Medical Drive, Singapore 117597, Singapore

**Keywords:** Genetics, Similarity, Power, Multi-locus, Association analysis, Rare variants, Collapsing, Weighting

## Abstract

**Background:**

As several rare genomic variants have been shown to affect common phenotypes, rare variants association analysis has received considerable attention. Several efficient association tests using genotype and phenotype similarity measures have been proposed in the literature. The major advantages of similarity-based tests are their ability to accommodate multiple types of DNA variations within one association test, and to account for the possible interaction within a region. However, not much work has been done to compare the performance of similarity-based tests on rare variants association scenarios, especially when applied with different rare variants pooling strategies.

**Results:**

Based on the population genetics simulations and analysis of a publicly-available sequencing data set, we compared the performance of four similarity-based tests and two rare variants pooling strategies. We showed that weighting approach outperforms collapsing under the presence of strong effect from rare variants and under the presence of moderate effect from common variants, whereas collapsing of rare variants is preferable when common variants possess a strong effect. We also demonstrated that the difference in statistical power between the two pooling strategies may be substantial. The results also highlighted consistently high power of two similarity-based approaches when applied with an appropriate pooling strategy.

**Conclusions:**

Population genetics simulations and sequencing data set analysis showed high power of two similarity-based tests and a substantial difference in power between the two pooling strategies.

## Background

Although genome-wide association studies (GWAS) have identified many common single nucleotide polymorphisms (SNPs) associated with common diseases ( http://www.genome.gov/gwastudies/), these common variants explain only a small fraction of the phenotypic variance attributable to genetic factors [1,2]. Recently, the scientific community has devoted a lot of attention to the analysis of rare variants, with the hope of finding the missing heritability. Indeed, there is growing evidence that rare variants are associated with some complex traits [3-6]. Therefore, research in the area of rare variants has a high potential to discover unknown associations of genomic regions with complex phenotypes. Numerous methodologies have been developed to test association of multiple rare variants within a region with a phenotype [7-11].

Measures of genotype similarity have been the basis of many proposed statistical tests. The idea of similarity-based tests is to consider the relationship between genotypic and phenotypic similarities (similarity here roughly refers to a measure of closeness of two genotypes or phenotypes). Similarity-based tests are motivated by the fact that haplotypes carrying the same causal mutation are more related compared with those without causal mutations; so, case haplotypes are expected to share longer stretches of DNA identical by descent [12]. One of the major advantages of similarity-based tests is the ability to accommodate multiple types of DNA variations (SNPs, insertions and deletions, CNV) observed within a region, given flexibility in the choice of similarity measures between two sequences [13]. Another issue that similarity-based tests address is the possible interaction of different variants within a region, which is potentially accounted for by considering multi-site similarity measures [14]. For unrelated individuals, similarity measures have been incorporated within a framework of single SNP analysis of variance [15], multiple regression [16], U-statistic [17] and distance-based regression [14]. Methods based on genotype similarity include the following: sequence kernel association test (SKAT) [11]; kernel-based association test (KBAT) [18], multivariate distance matrix regression test (MDMR) [19]; and aggregate U-test [20]. However, so far, no attempts have been made to evaluate the performance of similarity-based tests on rare variants association scenarios when common variants are included into or excluded from the analysis. Even though many non-causal common SNPs are removed by considering only rare variants, it is unclear if consideration of fewer variants would be sufficient to compensate for the loss of association signal from common SNPs. Also, when both rare and common variants are included into the analysis, it is of interest to evaluate the change in the performance of the tests when rare variants have lower effect sizes than common SNPs. Additionally, statistical tests may utilize different pooling strategies for rare variants, e.g. weighting or collapsing. Given the choice, it is unclear which pooling strategy is the best to be applied with similarity-based tests.

In this article, we compared the performance of the four similarity-based tests (SKAT, KBAT, MDMR and a modified U-test proposed by Schaid et al. [17]) applied with two popular rare variants pooling strategies (weighting and collapsing). The comparison was performed based on population-genetics simulations under four different disease models and the GAW17 sequencing data set. The results highlighted that, under the presence of strong rare variants association signal and moderate association of common variants, weighting may be a much better strategy than collapsing, whereas collapsing tends to outperform weighting when common variants possess a strong effect. Moreover, we discovered that the magnitude of the difference in power among similarity-based methods, when applied with weighting and collapsing strategies, may be very high, sometimes over 50%. Under the strong effect size of rare variants when common variants were excluded from the analysis, we observed better performance of collapsing strategy and lower power of weighting pooling strategy. Also, when the appropriate pooling strategy is applied, both SKAT and KBAT showed consistently high power among all the four similarity-based tests compared here.

## Results

### Population genetic simulations

For each test, 1000 permutations were performed to assess the significance of association. To make sure the empirical type-1 error is controlled, we ran the analysis of simulated data under the null model. As can be seen from Additional file 1: Table S1, the type-1 error was well controlled by using the permutation procedure to estimate the significance level. It is noticeable that for “Risk Rare” scenario when weighting pooling strategy is applied and for “Risk Common” scenario the estimates of type-1 error are below 0.05. This suggests that in these cases the methods show slightly conservative behavior. The double-sided 99% confidence interval for the type-1 error estimate is approximately 0.033–0.67. This can be derived from the normal approximation, given that the estimate of type-1 error rate is distributed as an observed probability of success for a binomial random variable with a success probability of 0.05 under no inflation of type-1 error and the sample size of 1000, which is the number of data replicates. As can be seen, the empirical type-1 error estimates for population genetics simulations were within the 99% confidence interval.

**Figure 1:**
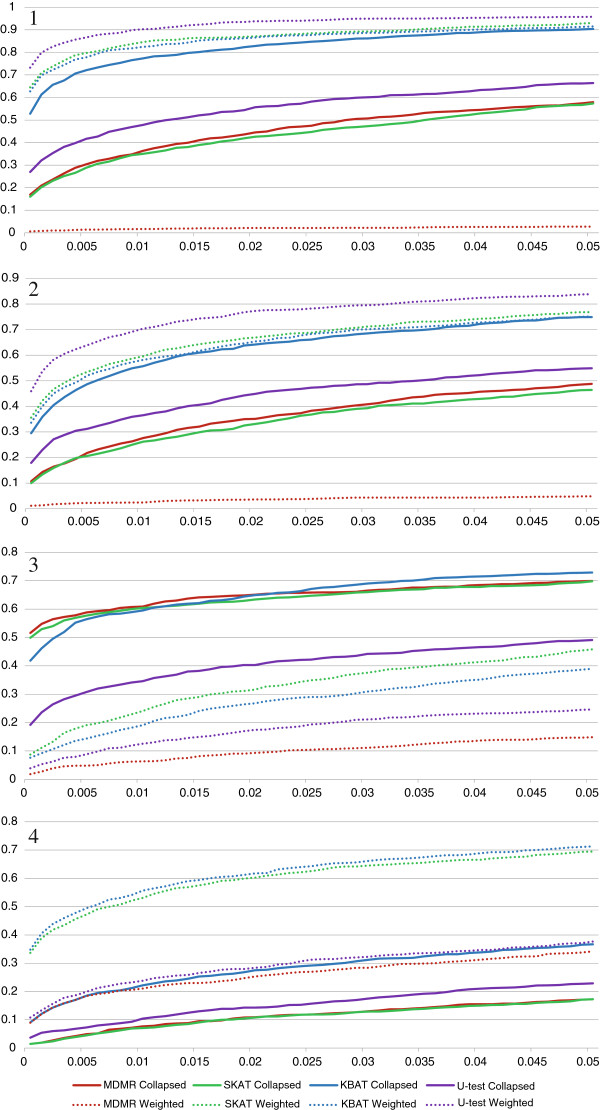
**Power as a function of significance level for the four similarity-based tests and two rare variants pooling strategies.** Panel 1: “Risk Rare” Scenario; Panel 2: “Risk Both” Scenario; Panel 3: “Risk Common” Scenario; Panel 4: “Mixed Rare” Scenario.

Figure 1 shows the power of the four tests with collapsing and weighting pooling strategies under different association scenarios. As can be seen from Panel 1 (“Risk Rare” scenario), the power of the tests was in the following order: for collapsing MDMR performed no worse than SKAT but no better than U-test, which in turn had lower power compared to KBAT; for weighting, MDMR performed worse than KBAT, and KBAT was no more powerful than SKAT, whereas U-test was the most powerful among all the four tests considered. Also, weighting increased the power of all the tests, except for MDMR. The same situation was observed when a weak association signal from common variants was introduced, together with weaker signal from rare variants (“Risk Both” scenario in Panel 2 of Figure 1). The performance of the tests when rare variants had lower effect compared with common variants (“Risk Common” scenario) is presented in Panel 3 of Figure 1. As can be seen, the pattern is different from those observed for the previous two scenarios. For all the tests, the collapsing strategy performed better than weighting. These results suggest that weighting outperforms collapsing when strong rare variants association is present; however, when common variants explain a significant portion of phenotype variability, collapsing is more advantageous since the weighting scheme undermines the signal from common variants.

Finally, we investigated the performance of the tests in the “Mixed Rare” scenario which incorporated both risk and protective variants within a region (Figure 1, Panel 4). As expected, collapsing underperformed weighting pooling strategy because collapsing risk and protective variants annihilates the association signal. Overall, the simulation results highlighted the consistently high power of KBAT and SKAT when the appropriate rare variants pooling strategy was applied (namely, collapsing for “Risk Common” scenario, and weighting for other scenarios). Although these two tests had slightly lower power compared to the U-test for “Risk Rare” and “Risk Both” scenarios, they had much higher power for the other two scenarios. Additionally, we calculated the maximum absolute power difference over the type-1 error rate for each test and phenotype scenario. As can be seen from Table 1, the maximum absolute power difference was substantial, and ranged from as low as 10% to as high as 55%. The average maximum absolute power difference for the considered tests across the phenotype models were 39.8%, 43.9%, 25.8% and 31.8% for MDMR, SKAT, KBAT and U-test respectively. This result shows the extreme importance of choosing the right rare variants pooling strategy for different disease models. As in our simulations no adjustment for population stratification was made, we analyzed the data using another popular similarity measure: identity-by-state [14]. The results were similar to those obtained for exponential similarity (Additional file 2).

**Table 1:** The maximum absolute difference in power (over the type-1 error rate) between weighting and collapsing pooling strategies for different tests and phenotype scenarios in population genetics simulations

**Scenario/Test**	**MDMR**	**SKAT**	**KBAT**	**U-Test**
**Risk Rare**	0.466	0.472	0.157	0.511
**Risk Both**	0.395	0.29	0.094	0.379
**Risk Common**	0.551	0.479	0.388	0.235
**Mixed Rare**	0.18	0.516	0.393	0.148

**Figure 2:**
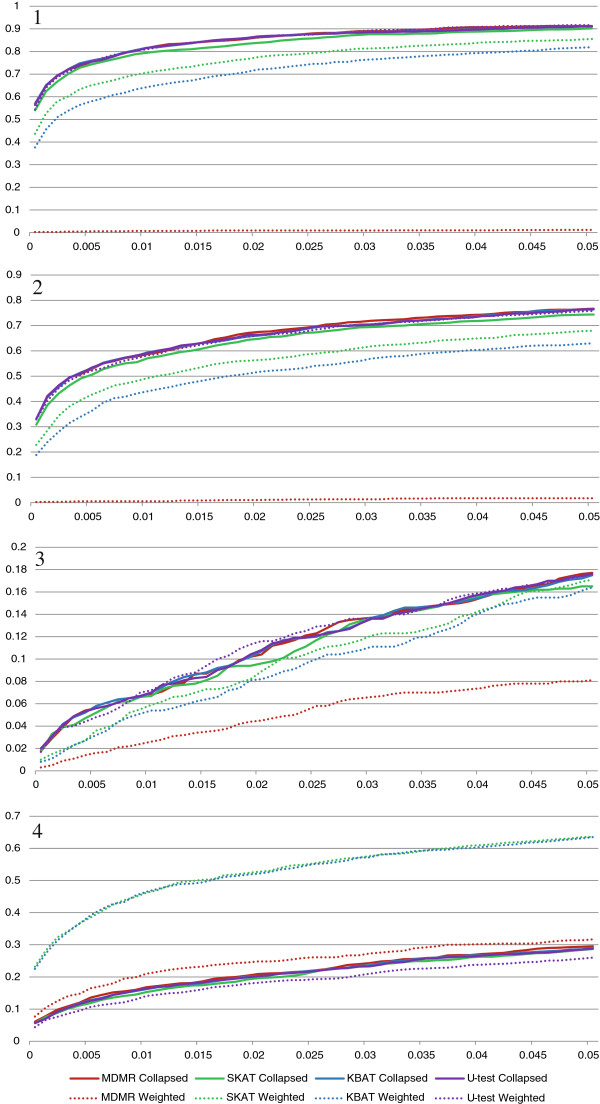
**Power as a function of significance level for the four similarity-based tests and two rare variants pooling strategies when common variants are excluded from the analysis.** Panel 1: “Risk Rare” Scenario; Panel 2: “Risk Both” Scenario; Panel 3: “Risk Common” Scenario; Panel 4: “Mixed Rare” Scenario.

We also analyzed the simulated data after excluding all common variants defined as those with MAF > 1% (Figure 2). Since all common variants were excluded, association tests under collapsing were performed only on a collapsed super-locus. In contrast to the previous results, for “Risk Rare” and “Risk Both” scenarios, collapsing performed better and weighting performed worse. For the collapsing strategy, the reduction in the number of SNPs was beneficial despite the loss of association signal from the excluded common SNPs. However, for the weighting pooling strategy, the loss of some of the causal variants with MAF above 1% lowered the statistical power of the tests. Hence, the results suggest that under strong rare variants effect size in one direction, one should prefer collapsing to weighting when common variants are excluded from an association test. For the “Risk Common” scenario, the power of all the tests and all pooling strategies was lower, as the strong association signal from common variants had been removed. For the “Mixed Rare” scenario, we observed that the results were similar to those depicted in Figure 1. Also, it is notable that across the four scenarios, the performances of the four tests were very similar under the collapsing strategy. This suggests that for a single SNP analysis, the power of the four tests is very similar to one another.

### GAW17 data set

The GAW17 data set is a large scale exome sequencing data set with genotypes from the 1000 Genomes Project ( http://www.1000genomes.org). The dataset consists of 697 unrelated individuals from six populations (Centre d'Etude du Polymorphisme Humain (CEPH) samples, Tuscan, Chinese, Japanese, Yoruba and Luhya). The complex phenotype model incorporates environmental covariates (age, sex and smoking status) and both common and rare causal SNPs from genes in particular pathways. Totally 200 replicates of several quantitative traits and case–control status were simulated under the phenotype model. A more detailed description of the simulations can be found in Almasy et al. [21].

We performed an association analysis of causal genes that affect two quantitative traits, Q_1_ and Q_2_, and a dichotomous trait, *D*. Adjustment for covariates was done in a similar way as in Jiang et al. [22]. Let *G* be the genotype matrix, *Q*_*j,*_*j* = 1, 2 are vectors of two quantitative traits, *E*_*i*_*i* = 1, 2, 3, are vectors of covariates (age, sex and smoking status, respectively), and *R* is the matrix of ten principal components of genotype matrix obtained using the software Eigenstat [23]. The corrected genotype, phenotypes and covariates are $\overset{⌣}{G}=G-RR^{T}D$, $\tilde{Q}j=Q_{j}-RR^{T}Q_{j},\;j=1,2$, $\tilde{D}=D-RR^{T}D$ and *˜E*_*i*_ *= E*_*i*_*-RR*^*T*^*E*_*i*_*, i* = 1,2,3. Next, covariates are regressed out of adjusted phenotypes using the regression models:

(1) $$\begin{array}{c} {\tilde{Q}}_{1}=a_{0}+\sum_{i=1}^{3}a_{i}{\tilde{E}}_{i}+\varepsilon_{1};{\tilde{Q}}_{2}=b_{0}\\ \;+\sum_{i=1}^{3}b_{i}{\tilde{E}}_{i}+\varepsilon_{2};\tilde{D}=C_{0}+\sum_{i=1}^{3}c_{i}\tilde{E}+\varepsilon_{3} \end{array}$$

The residuals from the regression models (1) were dichotomized (upper 30% of the observed distribution were declared cases, while the others were controls) and tested for association with adjusted genotype *Ğ* of the causal genes. The type-1 error was set at 0.05, and 1000 permutations were performed for each of the 200 phenotype replicates to assess the power. To assess the empirical type-1 error rate for all the statistical tests, we ran the analysis with randomly permuted adjusted phenotypes obtained from the regressions (1). The resulting type-1 error rates are presented in Additional files 3 and 4. The double-sided 99% confidence interval for the type-1 error estimate is approximately 0.01–0.09. This can be derived from the normal approximation, given that the estimate of type-1 error rate is distributed as an observed probability of success for a binomial random variable with a success probability of 0.05 under no inflation of type-1 error and the sample size of 200, which is the number of phenotype replicates. As can be seen, the empirical type-1 error for GAW17 data was within the 99% confidence interval.

**Figure 3:**
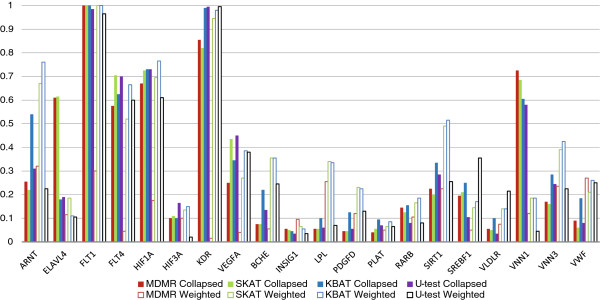
Power to identify association with dichotomized adjusted quantitative trait in GAW17 data set for causal genes (ARNT-VEGFC with Q1, and BCHE-VWF with Q2).

Figure 3 depicts the results of the analysis of the causal genes with the respective phenotypes (ARNT-VEGFC with Q1, and BCHE-VWF with Q2). For the majority of genes with rare causal variants, the weighting strategy, on average, performed better than collapsing (except for MDMR). For example, the weighing strategy resulted in substantial power improvement for the genes ARNT, SIRT1, VNN3 and VWF. All of these genes contained multiple causal rare variants with a moderate or high effect size. However, collapsing yielded a much higher power for ELAVL4 and VNN1 genes. Closer examination revealed that the two most common SNPs in the VNN1 gene were causal, whereas association with the ELAVL4 gene could be explained by association of the only two common SNPs that were non-causal. To show this, we analyzed these two common SNPs with the four similarity-based tests and found that the power to identify an association with a phenotype was as follows: MDMR – 0.6, SKAT – 0.585, KBAT – 0.135, U-test – 0.095. The results of the dichotomous phenotype analysis are presented in the Additional files 5 and 6. Among genes with maximum achieved power of greater than 40% for at least one of the tests, weighting was advantageous for the ARNT gene, whereas collapsing yielded higher power for FLT1 and PRKCA, which both contained common causal SNPs. So, the results of the GAW17 data set support the conclusion derived from population genetics simulations concerning pooling strategies. We also considered the maximum absolute difference in power between weighting and collapsing for each statistical test and each GAW17 phenotype (Q1, Q2 and dichotomous trait) over the respective causal genes. As can be seen from Table 2, the maximum absolute power difference ranged from 14.5% (U-test) to 84% (MDMR). The average maximum power differences across phenotypes were 73.8%, 45.6%, 35.6% and 40.5% for MDMR, SKAT, KBAT and U-test, respectively. This observation confirms the results obtained from our population genetics simulations and highlights the importance of the right choice of rare variants pooling strategy in sequencing association studies.

**Table 2:** The maximum absolute difference in power (over the respective causal genes) between weighting and collapsing pooling strategies for different tests and phenotypes in GAW17 data set

**Scenario/Test**	**MDMR**	**SKAT**	**KBAT**	**U-Test**
**Q1**	0.84 (KDR)	0.45 (ARNT)	0.22 (ARNT)	0.145 (HIF3A)
**Q2**	0.605 (VNN1)	0.5 (VNN1)	0.42 (VNN1)	0.535 (VNN1)
**Dichotomous**	0.77 (FLT1)	0.42 (PRKCA)	0.43 (PRKCA)	0.535 (FLT1)

The genes at which the maximum difference was achieved are in brackets.

## Discussion

In this article, we compared the performance of the four similarity-based tests together with two rare variants pooling strategies using population genetics simulations and the GAW17 real data set. The results suggest that weighting may be a much better strategy than collapsing under the assumption of strong effect from rare variants, and moderate or low effect from common variants. Collapsing, in turn, showed much better performance when common variants possessed a strong effect. The absolute power difference of a statistical test when applied with collapsing and weighting pooling strategies may be substantial. From our study, it follows that if researchers are inclined to believe in the association of rare variants within a region, weighted pooling should be applied with similarity-based tests, whereas collapsing is more appropriate if common variants are suspected to be associated with phenotype. Additionally, under strong rare variants effect size in one direction when common variants were excluded from the analysis, collapsing performed equally good or better than weighting. Finally, both SKAT and KBAT had consistently high power compared with other considered similarity-based tests when applied with the appropriate pooling strategy.

Recently, Basu and Pan [24] compared the performance of multiple statistical tests to identify an association with rare variants. The authors included SKAT with unweighted linear and quadratic kernels as one of the testing strategies. Based on the results, Basu and Pan [24] concluded that SKAT was powerful compared with other methods when only rare variants were considered. However, the authors found that the method lost its high power when neutral common variants were added. Our results suggest that using weighted kernels in SKAT may preserve high power to identify an association with rare variants even if multiple neutral common variants are included into the analysis. However, since we compared the performance of similarity-based tests, additional investigation is required to compare weighted similarity-based tests with other statistical strategies, including those considered in Basu and Pan [24].

From our results, the MDMR test does not seem to perform well when applied with weighting pooling strategy. To have a more detailed picture, we applied weighted MDMR test to the “Risk Rare” data sets with modified weights *w*_*l*_^*p*^, *l* = 1, … *L*, where the power value *p* varied from 0 to 1. So, *p = 1* corresponded to the beta weights applied in our study, whereas *p = 0* corresponded to the analysis without weights. Additional file 7 shows the power surface as a function of significance level and a value of *p*. It is clear that for all significance levels, the power of MDMR monotonically decreased with higher values of *p*, which corresponded to higher relative weights for rare variants. In the Additional file 1, we proved that when the number of cases equals the number of controls (like in our simulations), SKAT and MDMR test statistics are equivalent to the sum of, and the sum of squares of dissimilarities for all case–control pairs respectively. When weighting pooling strategy is applied, dissimilarity tends to be relatively large for pairs of individuals whose genotypes differ in multiple rare minor alleles. Squaring dissimilarity measure puts much more emphasis on pairs with a larger dissimilarity. Thus, the magnitude of the MDMR test statistic may be completely defined by the number of case–control pairs whose genotypes differ by at least two rare minor alleles. We suppose that pairs with a difference of one rare allele may not have sufficient dissimilarity to significantly influence the MDMR test statistic, which leads to a loss of power. To illustrate our reasoning, let us have two rare variants with only eight observed minor alleles each across 500 cases and 500 controls. To simplify the description, assume that individuals have either zero or one copy of a minor allele across the two variants. Also, we will use the equivalence of the MDMR test statistic to the sum of squared case–control dissimilarities. Consider the following cases under the null and alternative hypotheses, respectively: cases and controls have four minor alleles for each variant, and cases have all minor alleles. Under the alternative hypothesis, we have zero case–control pairs with a difference of two alleles across genotype, whereas under the null hypothesis, we have 32. However, under the alternative hypothesis, there are 16 × 500 case–control pairs with a difference of one minor allele, whereas under the null hypothesis, there are only 16 × (500–8). Now it becomes clear that if the dissimilarity of pairs of individuals with a difference of two alleles is large enough relative to the dissimilarity of pairs of individuals with a difference of only one allele, the MDMR test statistic may become lower compared to the null test statistic. The consideration above explains the low performance of MDMR with weighted similarity and the fact that for the “Risk Rare” scenario, the power of MDMR test was below type-1 error rate.

One limitation of the current study is that the minimum significance level in population genetics simulations was 0.001. For genome-wide significance, the number of permutations needed to reliably estimate the significance is very large. This makes the comparison of the similarity-based tests at the genome-wide level prohibitive. In real GWAS studies, only few highly-significant genes will require a very large number of permutations to estimate *p*-values, as many genes with low or no association signal can be dropped out after a few thousand permutations. For highly significant genes, permutation procedure can be split into several parts and performed in parallel on a cluster.

## Conclusions

The performance of similarity-based tests applied with two rare variants pooling strategies was investigated. Population genetics simulations and sequencing data set analysis showed consistently high power of two similarity-based tests and a substantial difference in performance of the two rare variants pooling strategies.

## Methods

### Similarity-based tests

Assume that an association study involves *N* individuals (*N*^*A*^ cases and *N*^*U*^ controls), and within a genomic region *L* SNPs (both common and rare) were called. Let us denote the genotype matrix *G = {g*_*nl*_*, n = 1,…,N l = 1,…,L}* coded as minor allele counts, and the phenotype vector *Y = {y*_*n*_*, n = 1,…,N}* with the elements valued 1 for cases and −1 for controls (except when otherwise specified). The *N × N* similarity matrix is defined as *K* = {*s*(*g*_*n*_, *g*_*m*_)}_*n*,*m* = 1_^*N*^, where *g*_*n*_ is a multi-site vector of genotype {g_1n_,…,g_Ln_} for *n*th individual, and *s (x,y)* is a similarity function. There is a variety of examples of similarity functions published in statistical genetics literature (for examples, see Wu et al. [11], Wessel and Shork [14], and Mukhopadhyay et al. [25]). However, it is desirable for the similarity matrix *K* to be symmetric positive semi-definite as this is “the key to its use in many statistical analyses” [26]. Thus, we consider only those similarity measures that result in a positive definite similarity matrix. Examples of such similarity measures are the weighted linear kernel *s*(*g*_*n*_, *g*_*m*_) = ∑ _*l* = 1_^*L*^*w*_*l*_*g*_*nl*_*g*_*ml*_ for some fixed weights *w*_*l*_*,l = 1,…,L* the weighted quadratic kernel *s*(*g*_*n*_, *g*_*m*_) = (1 + ∑ _*l*_^*L*^*w*_*l*_*g*_*nl*_*g*_*ml*_)^2^, and the weighted IBS kernel *s*(*g*_*n*_, *g*_*m*_) = ∑ _*l* = 1_^*L*^*w*_*l*_(2 − |*g*_*nl*_ − *g*_*ml*_|). For our analysis, a popular exponential similarity measure [27] was used:

(2) $$s\left(g_{n},g_{m}\right)=\text{exp}\left\{-\sum_{l=1}^{L}{\left(g_{\mathrm{nl}}-g_{\mathrm{ml}}\right)}^{2}\right\}$$

The choice of similarity was motivated by the need to analyze quantitative genotype obtained as a result of population stratification adjustment (see Results section). As the exponential similarity is a function of the Euclidean distance between two multi-site genotypes, we consider this similarity to be more appropriate compared with, for example, another popular similarity measure, identity-by-state [17], which was designed to be applied to genotype codes.

### Weighting and collapsing

Here we consider the two major ways of rare variants pooling: weighting and collapsing. The SNP weights will be denoted as *w = {w*_*l*_*, l = 1,…,L}*. In general, they may be derived from observed minor allele frequency (MAF) or prior information. Here, we adopted the weights proposed by Wu et al. [11]: *w*_*l*_ = *Beta*(*maf*_*l*_; 1, 25)^2^, where *maf*_*l*_ is MAF of *l*th SNP, *Beta (a; b, c)* is the beta density distribution function with parameters *b* and *c* evaluated at point *a*. The weight function monotonically increases as MAF decreases, while, as noted by the authors, “putting decent nonzero weights for variants with MAF 1%–5%”. As noted by Wu et al. [11], setting *0* ≤ *b* ≤ *1* and *c* ≥ 1 allows for an increase in the weight of rare variants and a decrease in the weight of common variants. Thus, any values of parameters and from the specified range are acceptable. For the three tests (SKAT, MDMR and U-test), the weights are incorporated via the calculation of similarity matrix. Specifically, the weights incorporating similarity function s_w_ for the similarity matrix *K*_*w*_ is as follows:

(3) $$s_{w}\left(g_{n},g_{m}\right)=\text{exp}\left\{-\sum_{l=1}^{L}w_{l}{\left(g_{\mathrm{nl}}-g_{\mathrm{ml}}\right)}^{2}/\sum_{l=1}^{L}w_{l}\right\}$$

For the KBAT test statistic, the weights were incorporated differently (for details, see the description below) as the test does not use the multi-site genotype similarity.

The collapsing of rare variants was performed as described in Thalamuthu et al. [18], namely, by defining a super-locus *g*_*n(L+1)*_*,n = 1,…,N* as follows:

(4) $$g_{n\left(L+1\right)}=\text{min}\left\{2,\sum_{l:\mathrm{ma}f_{l}\leq 0.01}\left(g_{\mathrm{nl}}\right)\right\}$$

In general, this type of collapsing preserves more information than an indicator of at least one rare variant being present, as suggested by Li and Leal [28]. The collapsed genotype is treated as a new SNP (super-locus) *g*_*n(L+1)*_*,n = 1,…,N*, and a similarity matrix is constructed using common variants and the super-locus.

### Multivariate distance matrix regression (MDMR)

Let us denote *N x N* identity matrix *1*_*N*_ and a vector of 1 of size *N* as *1*_*N*_. Following Wessel and Schork [14], the test statistic is calculated according to the algorithm:

1. Phenotype projection matrix *H = Y(Y*^*T*^*Y)*^*-1*^*Y*^*T*^, where upper *T* denotes transposition.

2. Dissimilarity matrix *D* = {*d*_*ij*_}_*i*,*j* = 1_^*N*^ = 1_*N*_1_*N*_^*T*^ − *K*, where *K* is a similarity matrix defined above.

3. Gover’s centered matrix *G* = (1_*N*_ − 1_*N*_1_*N*_^*T*^/*N*)*A*(*I*_*N*_ − 1_*N*_1_*N*_^*T*^/*N*), where $A={\left\{-\frac{{\text{d}}_{\mathrm{ij}}^{2}}{2}\right\}}_{\text{i},\text{j}=1}^{\text{N}}$.

4. The test statistic *MDMR* = *tr*(*HGH*)/*tr*((*I*_*N*_ − *H*)*G*(*I*_*N*_ − *H*)), where *tr* is matrix trace.

Large values of the test statistic indicate a deviation from the null hypothesis of no association of a genotype with a phenotype.

### Sequence kernel association test (SKAT)

For this test, the phenotype vector *Y* = {y_n_,n = 1,…,N} is coded as 1 for cases and 0 for controls. The mean phenotype vector is defined as $\bar{Y}=N^{A}1_{N}/N$. Following Wu et al. [11], the test statistic is $T={\left(Y-\bar{Y}\right)}^{T}K(Y-\bar{Y})/2$. The *SKAT* test statistic under the null hypothesis is asymptotically distributed as the weighted sum of chi-squared random variables with one degree of freedom. Thus, the significance level can be assessed theoretically. Permutations can also be used to estimate the *p*-value empirically.

### U-test

The average similarity score between pairs of cases *U*_*1*_ and controls *U*_*0*_ is defined as follows:

(5) $$U_{1}=\sum_{\underset{n,m:Y_{n}=Y_{m}=1}{1\leq n\leq m\leq N}}2K_{\mathrm{nm}}/N^{A}\left(N^{A}-1\right)$$

(6) $$U_{0}=\sum_{\underset{n,m:Y_{n}=Y_{m}=-1}{1\leq n\leq m\leq N}}2K_{\mathrm{nm}}/N^{U}\left(N^{U}-1\right)$$

where *K*_*nm*_*, n, m = 1,…,N* are the elements of the *K* similarity matrix (*K* = {*K*_*nm*_}_*n*,*m* = 1_^*N*^). The U-test statistic is defined as *U* = (*U*_1_ − *U*_0_)^2^. Note that Shaid et al. [17] considered the weighted sum of the single SNP U-test statistics, where weights were derived from the asymptotic variance-covariance matrix of the U statistics vector. However, for the purpose of comparison of weighting and collapsing rare variants pooling methods, the statistic was modified as described above. The test statistic *U* is similar to the single SNP U-test statistic proposed by Shaid et al. [17], but it incorporates the similarity information across multiple variants within a region. Permutations need to be applied to assess the *p*-value.

### Kernel-based association test (KBAT)

Let us denote *K*_*l*_ = {(*K*_*l*_)_*nm*_}_*n*,*m* = 1_^*N*^ as a single SNPs similarity matrix for *l*th variant. Similar to the notations of the U-test subsection, *Ul*_*1*_ and *Ul*_*0*_ are the average similarity scores for pairs of cases and controls, respectively, calculated from *K*_*l*_, and let *U*_*l*_ = (*U*_*l*1_ + *U*_*l*0_)/2. Following Mukhopadhyay et al. [15], consider the within-group and between-group sum of squares:

(7) $$\begin{array}{c} \mathrm{WS}S_{l}=\sum_{\underset{n,mY_{n}=Y_{m}=1}{1\leq n\leq m\leq N}}{\left({\left(K_{l}\right)}_{\mathrm{nm}}-U_{l1}\right)}^{2}\\ \;+\sum_{\underset{n,mY_{n}=Y_{m}=-1}{1\leq n\leq m\leq N}}{\left({\left(K_{l}\right)}_{\mathrm{nm}}-U_{l0}\right)}^{2} \end{array}$$

(8) $$\begin{array}{c} \mathrm{BS}S_{l}=\frac{N^{A}\left(N^{A}-1\right){\left(U_{l}-U_{l1}\right)}^{2}}{2}\\ \;+\frac{N^{U}\left(N^{U}-1\right){\left(U_{l}-U_{l0}\right)}^{2}}{2} \end{array}$$

where the two groups are case-case and control-control pairs. The test statistic is *KBAT* = ∑ _*l* = 1_^*L*^*BSS*_*l*_/∑_*l* = 1_^*L*^*WSS*_*l*_. Since the test does not utilize the multi-site similarity matrix, but only single SNP matrices *K*_*l*_, the weighted test statistic *KBAT*_*W*_ = ∑ _*l* = 1_^*L*^*w*_*l*_*BSS*_*l*_/∑_*l* = 1_^*L*^*w*_*l*_*WSS*_*l*_ is used here. A large value of the *KBAT* statistic indicates a deviation from the null hypothesis. Permutations are used to assess the significance.

### Population genetics simulations

Population genetics simulations were performed based on the code provided by King et al. [29] with demographic parameters from Boyko et al. [30]. A total of 1000 data replicates were generated for each of the four phenotype models: “Risk Rare”, “Risk Both”, “Risk Common” and “Mixed Rare”. For a detailed description of the simulations, see Additional file 1.

## Competing interests

The authors declare that they have no competing interests.

## Authors’ contributions

SZ, AS and AT conceived the study. SZ and AT designed the experiments. SZ conducted the experiments and performed the analysis. SZ wrote the manuscript. SZ, AS and AT approved the manuscript.

## Supplementary Material

Additional file 1 Empirical type-1 error estimate for population genetics simulations (Table S1), detailed description of population genetics simulations, and considerations for possible reasons for MDMR power loss when applied with weighting pooling strategy. Click here for file

Additional file 2 **Power as a function of significance level for the four similarity-based tests with IBS kernels and two rare variants pooling strategies.** Panel 1: “Risk Rare” Scenario; Panel 2: “Risk Both” Scenario; Panel 3: “Risk Common” Scenario; Panel 4: “Mixed Rare” Scenario. Click here for file

Additional file 3 Empirical type-1 error rates for dichotomized adjusted quantitative phenotype in GAW17 data set at the theoretical level of 0.05 (ARNT-VEGFC with Q1, and BCHE-VWF with Q2). Click here for file

Additional file 4 Empirical type-1 error rates for dichotomized adjusted case–control status in GAW17 data set at the theoretical level of 0.05. Click here for file

Additional file 5 Power to identify an association with dichotomized adjusted case–control status in GAW17 data set for some of the causal genes. Click here for file

Additional file 6 Power to identify an association with dichotomized adjusted case–control status in GAW17 data set for some of the causal genes. Click here for file

Additional file 7 Impact of power value on MDMR test performance in a “Risk Rare” scenario. Click here for file
