# Supplementary material for: Comparison of similarity-based tests and pooling strategies for rare variants
Source: BMC Genomics. 2013 Jan 24;14:50. doi: 10.1186/1471-2164-14-50 (PMC3600007; doi:10.1186/1471-2164-14-50)
Supplement: Additional file 1 — Empirical type-1 error estimate for population genetics simulations (Table S1), detailed description of population genetics simulations, and considerations for possible reasons for MDMR power loss when applied with weighting pooling strategy. [file 1471-2164-14-50-S1.doc]

**Table S1 Empirical type-1 error rate for different disease scenarios, rare variants pooling strategies and statistical tests in population genetics simulations. The theoretical type-1 error rate is 0.05.**

| **Scenario** | **Collapsing** | | | | |  | | **Weighting** | | | |  |
| --- | --- | --- | --- | --- | --- | --- | --- | --- | --- | --- | --- | --- |
|  | **MDMR** | **SKAT** | **KBAT** | **U-test** |  | | **MDMR** | | **SKAT** | **KBAT** | **U-test** | |
| **Risk Rare** | 0.055 | 0.055 | 0.042 | 0.056 |  | | 0.042 | | 0.039 | 0.04 | 0.049 | |
| **Risk Both** | 0.05 | 0.051 | 0.046 | 0.044 |  | | 0.052 | | 0.039 | 0.04 | 0.054 | |
| **Risk Common** | 0.039 | 0.038 | 0.048 | 0.044 |  | | 0.048 | | 0.046 | 0.046 | 0.038 | |
| **Mixed Rare** | 0.053 | 0.045 | 0.055 | 0.051 |  | | 0.046 | | 0.055 | 0.051 | 0.044 | |

## Population genetics simulations

The genotype simulation is based on the population genetics history of a European population for the exonic regions of a gene ANGPTL4 (seven exonic regions of a total length of 1895 bps). To simulate genotypic variations within a European population, the program provided by King et al. (http://home.uchicago.edu/~crk8e) was used in the forward simulator SFS_CODE (http://sfscode.sourceforge.net). The program simultaneously implements the demographic history of three populations, African-Americans, Europeans and Asians, with the demographic and distribution fitness effect parameters from Boyko et al. and Gutenkunst et al. . The assumptions about the mutation rate (1.8/10^8^ per nucleotide per generation) and the local recombination rate (4 cM/mb; no recombination hotspots) were the same as in King et al. . Using the program described above, we generated 1000 haplotype pools, each containing 20 000 sampled “individuals” (40 000 chromosomes) from a European population. The same haplotype pools were used for simulations of all phenotype models. One haplotype pool was used for one data replicate.

To generate a data replicate, we sampled a pair of haplotypes randomly from a haplotype pool, and took the corresponding alleles from each position to get the marker genotypes. The phenotype was assigned based on the multi-site genotype using a linear logistic model whose parameters depended on the phenotype model. For our simulations, four phenotype models were implemented: “Risk Rare”, in which only risk-contributing rare variants are present within a region; “Risk Both”, in which both rare and common variants are causal, with rare ones having larger impact; “Risk Common”, in which both rare and common variants are causal with common ones having larger impact; and “Mixed Rare”, in which risk-contributing and protective rare variants are present. Causal SNPs were sampled randomly according to a scenario described in Table S2. For example, for a “Risk Rare” scenario, roughly 50% of the rare variants found within a haplotype pool were assigned to be causal and used in the phenotype model. For the purpose of presentation, let us denote as
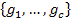
 the genotype of an “individual” at variants chosen to be causal and
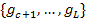
 genotype at other SNPs. Let
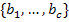
 be defined as the assigned odds ratio of causal variants (varies depending on scenario, see Table S2). The probability of a disease
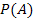
 for an “individual” is determined from:

|  | 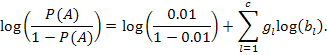 |  |
| --- | --- | --- |

The penetrance of wild type genotype was assumed to be 1% for all scenarios. The procedure of haplotype sampling and phenotype assigning proceeded until there were 500 cases and 500 controls. The average number of SNPs across data replicates for each phenotype model is presented in Table S3.

**Table S2** Summary of disease models for the four scenarios in population genetics simulation. Rare variants are defined as SNPs with observed MAF ≤ 1% in a haplotype pool, otherwise common.

| **Scenario** | **Causal variants choice description** | |  | **OR for each causal minor allele** | |
| --- | --- | --- | --- | --- | --- |
|  | **Rare** | **Common** |  | **Rare** | **Common** |
| **Risk Rare** | 50% of r. v.* in a pool | - |  | 4 | - |
| **Risk Both** | 50% of r. v. in a pool | 1 SNP |  | 3 | 1.2 |
| **Risk Common** | 50% of r. v. in a pool | 1 SNP |  | 1.5 | 2 |
| **Mixed Rare** | 25% of r. v. in a pool risk;  25% of r. v. in a pool protective; | - |  | 4 or 1/4 | - |

* r.v. stands for rare variants.

**Table S3 The** average number of causal and non-causal variants in data replicates by frequency category: rare and common. Rare variants are defined as SNPs with observed MAF ≤ 1% in a data replicate, otherwise common.

| **Scenario** | **Risk** | |  | **Protective** | |  | **Non-Causal** | |
| --- | --- | --- | --- | --- | --- | --- | --- | --- |
|  | **Rare** | **Common** |  | **Rare** | **Common** |  | **Rare** | **Common** |
| **Risk Rare** | 17.251 | 1.288 |  | - | - |  | 13.077 | 5.913 |
| **Risk Both** | 16.262 | 1.927 |  | - | - |  | 13.17 | 4.949 |
| **Risk Common** | 13.908 | 1.29 |  | - | - |  | 13.115 | 4.909 |
| **Mixed Rare** | 8.907 | 0.614 |  | 5.185 | 0.003 |  | 13.243 | 5.893 |

# Considerations for possible reason for MDMR power loss when applied with weighting pooling strategy

Let us adopt the notations as in the article and for simplicity assume equal number of cases and controls. Given that
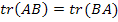
 for any real matrices
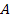
 and
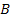
 of compatible dimensions and idenpotence of
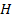
 matrix (
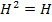
), it follows:


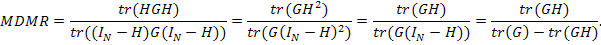


Since
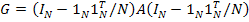
 we can rewrite:


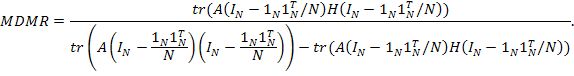


Given the matrix
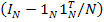
 is idempotent and
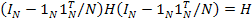
 when number of cases and controls are equal (as
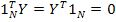
) it follows:


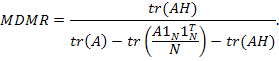


If we assume exponential similarity measure, the diagonal of similarity matrix
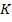
 is 1, so the diagonal of dissimilarity matrix
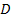
 as far as of matrix
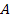
 is zero; thus,
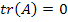
. Next,
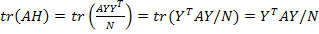
. So:


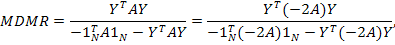


where we multiplied matrix
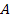
 by -2 to transfer to dissimilarity matrix
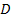
 as
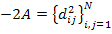
. Let us denote
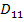
,
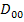
 and
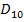
 as the sum of elements of matrix
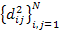
corresponding to all case-case, control-control and case-control pairs (a pair
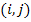
 is different from
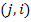
). So, we can rewrite the test statistic as:


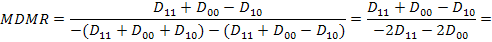


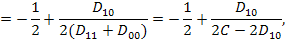


where
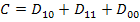
 is constant when permutation test is applied. Given that
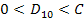
 and the function
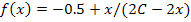
 is strictly monotonically increasing for
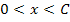
, the
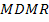
 test statistic is equivalent to
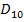
. From the definition of matrix
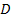
:


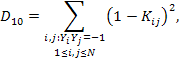


For the purpose of comparison let us transform the
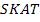
 test statistic. Taking into account the different phenotype coding for SKAT test:


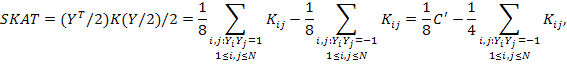


where
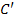
is the sum of all elements in the similarity matrix. Note,
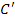
 is constant for permutation test. It is easy to show that the
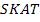
 test statistic is equivalent to:


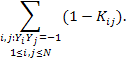


As can be seen,
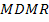
 and
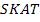
 test statistics are equivalent to a sum of squares and a sum of dissimilarities for all case-control pairs respectively.

**References**

1. King CR, Rathouz PJ, Nicolae DL: **An Evolutionary Framework for Association Testing in Resequencing Studies**. *PLoS Genet* 2010, **6**(11):e1001202.

2. Boyko AR, Williamson SH, Indap AR, Degenhardt JD, Hernandez RD, Lohmueller KE, Adams MD, Schmidt S, Sninsky JJ, Sunyaev SR *et al*: **Assessing the Evolutionary Impact of Amino Acid Mutations in the Human Genome**. *PLoS Genet* 2008, **4**(5):e1000083.

3. Gutenkunst RN, Hernandez RD, Williamson SH, Bustamante CD: **Inferring the Joint Demographic History of Multiple Populations from Multidimensional SNP Frequency Data**. *PLoS Genet* 2009, **5**(10):e1000695.
