# Supplementary figures and images for: Comparison of similarity-based tests and pooling strategies for rare variants
Source: BMC Genomics. 2013 Jan 24;14:50. doi: 10.1186/1471-2164-14-50 (PMC3600007; doi:10.1186/1471-2164-14-50)

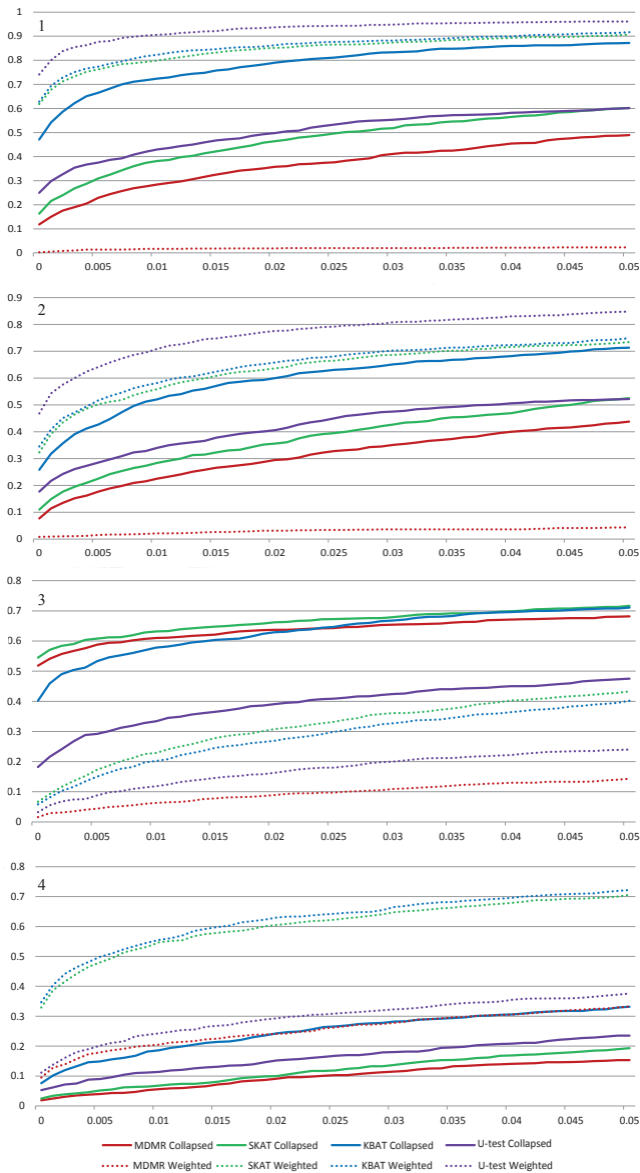

Supplement: Additional file 2 — Power as a function of significance level for the four similarity-based tests with IBS kernels and two rare variants pooling strategies. Panel 1: “Risk Rare” Scenario; Panel 2: “Risk Both” Scenario; Panel 3: “Risk Common” Scenario; Panel 4: “Mixed Rare” Scenario. [file 1471-2164-14-50-S2.pdf]

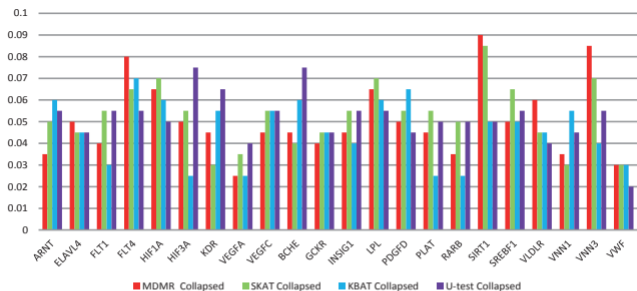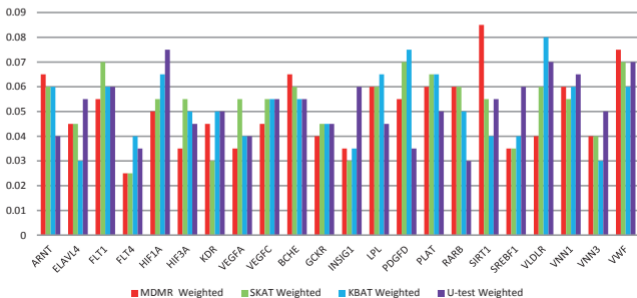

Supplement: Additional file 3 — Empirical type-1 error rates for dichotomized adjusted quantitative phenotype in GAW17 data set at the theoretical level of 0.05 (ARNT-VEGFC with Q1, and BCHE-VWF with Q2). [file 1471-2164-14-50-S3.pdf]

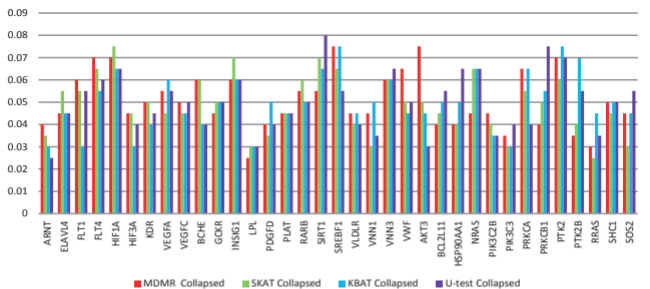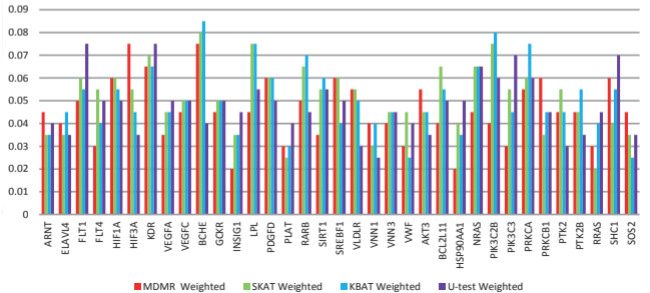

Supplement: Additional file 4 — Empirical type-1 error rates for dichotomized adjusted case–control status in GAW17 data set at the theoretical level of 0.05. [file 1471-2164-14-50-S4.pdf]

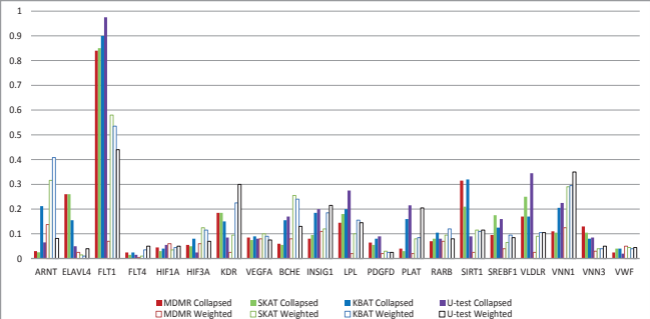

Supplement: Additional file 5 — Power to identify an association with dichotomized adjusted case–control status in GAW17 data set for some of the causal genes. [file 1471-2164-14-50-S5.pdf]

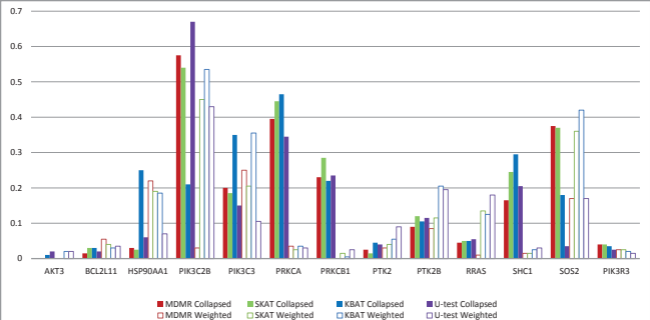

Supplement: Additional file 6 — Power to identify an association with dichotomized adjusted case–control status in GAW17 data set for some of the causal genes. [file 1471-2164-14-50-S6.pdf]

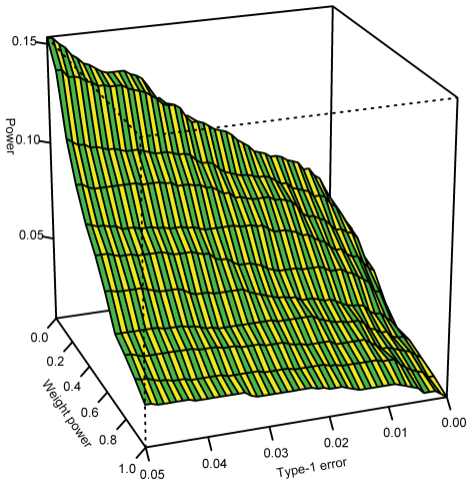

Supplement: Additional file 7 — Impact of power value on MDMR test performance in a “Risk Rare” scenario. [file 1471-2164-14-50-S7.pdf]
